# Hydrogen Sulfide—a potent multichannel anti-arrhythmic drug

**DOI:** 10.4103/0975-3583.59984

**Published:** 2010

**Authors:** Guang-Zhen Zhong

**Affiliations:** *Department of Cardiology, Beijing Chaoyang Hospital, Capital Medical University, Beijing, P.R. China 100020 zhongguangzhen@hotmail.com*

## Abstract

—Arrhythmia is a big headache for cardiologist for a long time. A new antiarrythmic drug with good effects and few side effects should be explored. Hydrogen sulfide, a newly found gaseous transmitter, was found to have multi-effects on a variety of ion channels. It may be a promising antiarrythmic drug. This article reviews the different effects of hydrogen sulfide on different ion channels.

Ion channels initiate and conduct electrical activity in the heart. The channels open and close in response to changes in membrane potential, and allow ionic permeation across the plasma membrane. The idea that defective ion channels might be responsible for cardiac arrhythmia has received indirect support from the anti- or proarrhythmic actions of a host of drugs and toxins that are specific for Na^+^, Ca^2+^ and K^+^ channels. With the progress of the clinical trials, it is widely understood that drugs having multi-effects on various ion channels may be the promising antiarrhythmic drug

## Antiarrhythmic drugs today

Recently, cardiologists are more interested in devices and ablation techniques for arrhythmia treatment than in pharmacological treatment. However, pharmacological treatment is still very important especially in prevention of atrial fibrillation and sudden cardiac death.

It is well known that present antiarrhythmic drugs are not ideal. They induce another arrhythmia when treating one arrhythmia or increased the total death rate although inhibited arrhythmia. Therefore, new antiarrhythmic drugs are still welcome.

To date, the most popular antiarrhythmic drug classification is the system developed by Singh and Vaughan Williams^1^ in the early 1970s and subsequently modified by Singh and Hauswirth and by Harrison.^2^ In this system, antiarrhythmic drugs were divided into class I for sodium-channel blockade (with subclasses IA, IB and IC), class II for adrenergic antagonism, class III for action-potential prolongation, and class IV for calcium-channel blockade. Among these drugs, since the publication of studies demonstrating that certain drugs may increase mortality in highrisk post-infarction patients,^3^ basic science and clinical studies have focused on Class III antiarrhythmic drugs. However, Class III drugs prolong repolarization and cardiac refractoriness, and are sometimes associated with potentially lethal Torsades de pointes. Amiodarone, a multichannel blocker, may be the exception to this observation. Therefore, both cardiologists and pharmacologists are turning their interests towards developing new multichannel blocker, since amiodarone nevertheless fails to reduce total mortality compared with placebo in high-risk patients following myocardial infarction.^4^

## Hydrogen sulfide—a potent multichannel acting antiarrhythmic drug -

Recent studies^5^,^6^ showed that hydrogen sulfide (H_2_S), new gaseous transmitter, acted on several cardiac ion channels, inhibited arrhythmia and prevented ischemia-reperfusion injury. It is reasonable to think of it as a potent promising multichannel acting antiarrhythmic drug.

H_2_S has been best known for decades as the toxic gas or ‘gas of rotten eggs’. It has been founded as a new gaseous transmitter only in recent years.^7^ And from 2003, H_2_S has been widely studied in different pathological animals. Most of these studies showed that H_2_S exerts its function through acting on different ion channels.^8^,^9^

## Effects of H_2_S on ATP dependent potassium channel (K_ATP_)

The first study^10^ about the action of H_2_S on ion channels was from Zhao’s study, and they studied the effects of H_2_S on K_ATP_. They proved the opening effect of H_2_S on K_ATP_ channel through three different levels. First, they found blood pressure decreasing effect of H_2_S was antagonized by blockade of K_ATP_ channels. Then their results showed H_2_S relaxed rat aortic in vitro in a K_ATP_ channel-dependent manner. Finally, they proved with patch clamp technique that H_2_S increased K_ATP_ channel currents. After that, Geng et al^11^ found H_2_S has negative inotropic effect through opening K_ATP_ channel. Zhang and colleagues^5^ did an even more interesting study. They made an ischemia-reperfusion model in vitro by Langendorff apparatus. They found H_2_S had a cardioprotective effect against ischemia-reperfusion injury. Moreover, they found H_2_S decreased arrhythmia score significantly in this model. Through patch-clamp technique, they also found that cardioprotective mechanism of H_2_S was opening of K_ATP_ channel. Zhang’s results challenged the traditional point of view, i.e. shortening of repolarization of cardiac cells due to opening of potassium channel may shorten the refractory period and induce arrhythmia. This may be explained by the following mechanisms: 1) Although H_2_S shortened refractory period of ventricular myocardial cells, it prolonged the effective refractory period relatively; 2) H2S has greater effects on other ion channels.

To further explore the mechanism of H_2_S on K_ATP_, Zhang et al.^12^ studied the effect of H_2_ S on gene and protein expression of K_ATP_ (SUR2B mRNA and Kir 6.1mRNA). Their results showed that H_2_S increased the gene expression of K_ATP_ as well as the protein expression.

## Effects of H_2_S on Calcium channel

The first study that may involve L-Calcium channel is from Geng’s work.^11^ Both in vivo and in vitro experiments in this study proved that H_2_S had a negative inotropic effect on rat heart. This may suggest the effect of H_2_S on L-calcium channel, although the authors did not perform any related experiments.

As researchers focused on the effect of H_2_S on K_ATP_ channel, Xiao et al^13^ noticed another channel—L-calcium channel was also involved in the acting mechanism of H_2_S. They studied the effect of H_2_S on the functional curve of baroreflex. The results showed H_2_S facilitated the carotid sinus baroreceptor. Pretreatment with a K_ATP_ channel blocker, abolished the above effects while pretreatment with Bay K8644 (an agonist of calcium channels) eliminated the effect of H_2_S on carotid sinus baroreceptor. Therefore, they concluded that H_2_S opened K_ATP_ channels and further closed the calcium channels.

The above study is on carotid sinus baroreceptor, which may be quite different from myocardial cells. Yet, Xu and colleagues^14^ studied the effect of H_2_S on L-calcium channel on guinea pig papillary muscles. Their data showed that pretreatment with L-type Ca^2+^ channel agonist Bay K8644 partially blocked the effects of NaHS (a H_2_S donor), which suggested that the reduction of calcium influx may also contribute to the effects of H_2_S. Furthermore, they found that pretreatment with Ca^2+^ -free K-H solution containing Glibenclamide completely blocked the effects of H_2_S, which indicated that the effects of H_2_S guinea pig papillary are due to the changes of potassium and calcium currents.

Step further, Sun et al^15^ studied the effects of H_2_S on L-Calcium channel directly, and stated definitely that Hydrogen sulphide is an inhibitor of L-type calcium channels. Sun and colleagues found a concentration-dependent inhibition of peak I_Ca.L_ on cardiomyocytes by NaHS (a H_2_S donor). It becomes clear that H_2_S has an inhibitory effect on L-Calcium channel. However, the mechanism needs further study.

Although, no study was reported about the H_2_S on T type Calcium channel on cardiomyocytes, Nagasawa et al.^16^ studied the effect of H_2_S on T-type calcium channels on NG 108–15 cells (which may be differentiated into neuronal cells). Their study showed H_2_ S could stimulate the T-type calcium channels. Therefore, it is reasonable to think of H_2_S as having an effect on T-type calcium channels, which needs further exploration.

## Effects of H_2_S on chloride channel

Chloride channels are important for setting cell resting membrane potential and maintaining proper cell volume. These channels conduct Cl^−^ as well as other anions such as HCO^3−^, I^−^, SCN^−^, and NO^3−^. Chloride channels display a variety of important physiological and cellular roles that include regulation of pH, volume homeostasis, organic solute transport, cell migration, cell proliferation and differentiation. Recent studies have identified several chloride (Cl^−^) channel genes in the heart, including CFTR, ClC^−2^, ClC^−3^, CLCA, Bestrophin, and TMEM16A. It has been shown that Cl^−^ channels may contribute to cardiac arrhythmogenesis, myocardial hypertrophy and heart failure, and cardioprotection against ischaemia–reperfusion.^17^

Since the reason above, we are concerned of the effect of H_2_S on chloride channel. The effect is reported on June 2009 by Malekova et al.^9^, where they derived single chloride channels from the rat heart lysosomal vesicles incorporated into a bilayer lipid membrane. Their results showed that H_2_S inhibited the chloride channels by decreasing the channel open probability in a concentration-dependent manner. Since they incorporated the single chloride channels into the bilayer lipid membrane, they measured the IC_50_ for tans and the cis sides, respectively and got the different results (42uM vs. 75uM).

## Beneficial cardiac effects from H_2_S

With the results of the famous CAST clinical trial,^3^ people realized that benefits for the patient’s prognosis is more important than antiarrhythmia itself. So the effect of H_2_S on the whole heart may be more important.

The effect on the whole heart was first described in Geng’s study^18^ Their results showed that administration of exogenous H_2_S effectively protects cardiomyocytes and contractile activity from isoproterenol injury. Then, Zhang et al^5^ isolated rat heart, then made an ischemia-reperfusion model with langendorff apparatus. The results showed that the treatment of hearts with a H_2_S donor during reperfusion resulted in a significant improvement in heart function and the arrhythmia scores was also improved. Furthermore, Sivarajah et al.^19^ found in the ischemia-reperfusion model that the antiapoptotic effect of NaHS (H_2_S donor) may be in part due to the opening of the putative mitochondrial adenosine triphosphate-sensitive potassium channels.

## CONCLUSION

In summary, H_2_S has various effects on various ion channels from cardiomyocytes. Not only can it improves heart arrhythmia score, but also can protect heart from ischemia-reperfusion injury and has beneficial effects on the heart function. We have reasons to think of it as a promising antiarrhythmic drug. Since it was a newly found endogenous gaseous signaling molecule, the risk, safety issues, dosage, proposed delivery methods and so on are therefore a big field to be explored.
